# Supplementary material for: Diversity and functional specialization of oyster immune cells uncovered by integrative single-cell level investigations
Source: eLife. 2025 May 9;13:RP102622. doi: 10.7554/eLife.102622 (PMC12064177; doi:10.7554/eLife.102622)
Supplement: Peer review history

# Editor report: eLife Assessment

**DOI:** 10.7554/eLife.102622.4.sa0

**Authors:** Bruno Lemaitre

**Affiliations:** École Polytechnique Fédérale de Lausanne Switzerland

**Keywords:** Convincing, Important

The manuscript by de La Forest Divonne et al. offers an **important** and detailed exploration of the immune cells in the oyster Crassostrea gigas, by correlating distinct hemocyte morphotypes with specific single-cell transcriptional profiles. The evidence supporting the conclusion is **convincing**, deriving from the comprehensive dataset that not only captures unicellular diversity but also associates these cells with distinct immune roles, making it an invaluable resource for the broader research community.

# Referee report: Reviewer #1 (Public review)

**DOI:** 10.7554/eLife.102622.4.sa1

Summary

In this manuscript, De La Forest Divonne et al. build a repertory of hemocytes from adult Pacific oysters combining scRNAseq data with cytologic and biochemical analyses. Three categories of hemocytes were described previously in this species (i.e. blast, hyalinocyte and granulocytes). Based on scRNAseq data, the authors identified 7 hemocyte clusters presenting distinct transcriptional signatures. Using Kegg pathway enrichment and RBGOA, the authors determined the main molecular features of the clusters. In parallel, using cytologic markers, the authors classified 7 populations of hemocytes (i.e. ML, H, BBL, ABL, SGC, BGC, and VC) presenting distinct sizes, nucleus sizes, acidophilic/basophilic, presence of pseudopods, cytoplasm/nucleus ratio and presence of granules. Then, the authors compared the phenotypic features with potential transcriptional signatures seen in the scRNAseq. The hemocytes were separated in a density gradient to enrich for specific subpopulations. The cell composition of each cell fraction was determined using cytologic markers and the cell fractions were analysed by quantitative PCR targeting major cluster markers (two per cluster). With this approach, the authors could assign cluster 7 to VC, cluster 2 to H, and cluster 3 to SGC. The other clusters did not show a clear association with this experimental approach. Using phagocytic assays, ROS, and copper monitoring, the authors showed that ML and SGC are phagocytic, ML produces ROS, and SGC and BGC accumulate copper. Then with the density gradient/qPCR approach, the authors identified the populations expressing anti-microbial peptides (ABL, BBL, and H). At last, the authors used Monocle to predict differentiation trajectories for each subgroup of hemocytes using cluster 4 as the progenitor subpopulation.

The manuscript provides a comprehensive characterisation of the diversity of circulating immune cells found in Pacific oysters.

Strengths

The combination of scRNAseq, cytologic markers and gradient based hemocyte sorting offers an integrative view of the immune cell diversity.

Hemocytes represent a very plastic cell population that has key roles in homeostatic and challenged conditions. Grasping the molecular features of these cells at the single-cell level will help understand their biology.

This type of study may help elucidate the diversification of immune cells in comparative studies and evolutionary immunology.

Weaknesses

Several figures show inconsistency leading to erroneous conclusions and some conclusions are poorly supported. Moreover, the manuscript remains highly descriptive with limited comparison with the available literature.

Comments on revisions:

The authors replied to most comments.

# Author comment: Author response

**DOI:** 10.7554/eLife.102622.4.sa2

**Authors:** Sebastien De La Forest Divonne, Juliette Pouzadoux, Oceane Romatif, Caroline Montagnani, Guillaume Mitta, Delphine Destoumieux-Garzón, Benjamin Gourbal, Guillaume M Charriere, Emmanuel Vignal

**Affiliations:** University of Perpignan Montpellier France; French Research Institute for Exploitation of the Sea Montpellier France; French Research Institute for Exploitation of the Sea Montpellier France; Ifremer Montpellier France; French Research Institute for Exploitation of the Sea Tahiti France; Univ Montpellier Montpellier France; UMR 5244 IHPE CNRS Perpignan France; University of Montpellier Montpellier France; University of Montpellier Montpellier France

The following is the authors’ response to the previous reviews

**Reviewer #1 (Recommendations for the authors):**

Major comments

(1) Line 201: The threshold of 0.25 was maintained to select enriched genes, which minimize the value of the GO term enrichment analyses. It may notably explain why the term phagosome is enriched in cluster 7, while experimental data indicate that cluster 7 is not phagocytic. In addition, the authors mentioned in the 1st response to reviewer that they would include DotPlot to illustrate the specificity of the genes corresponding to the main GO terms. This should notably include the ribosomal genes found enriched in cluster 4, which constitute the basis used by the authors to call cluster 4 the progenitor cluster.

We appreciate the reviewer’s concern regarding our chosen log2FC threshold (0.25) for GO term enrichment. To assess the robustness of our approach, we tested more stringent thresholds (e.g., 0.5) and verified that our overall interpretations remain consistent. However, we acknowledge that certain GO terms, such as phagosome, may appear in clusters that are not primarily phagocytic. This is likely due to the fact that genes involved in vesicle trafficking, endo-lysosomal compartments and intracellular degradation processes overlap with those classically associated with phagocytosis.

Therefore, the KEGG-based enrichment of phagosome in cluster 7 does not necessarily imply active phagocytosis but could instead reflect these alternative vesicular processes. As we show, cluster 7 correspond to vesicular cells, and as seen in cytology we named these cells after their very high content of vesicular structures. As functional annotation based solely on transcriptomic data can sometimes lead to overinterpretations, we emphasize the importance of biological validation, which we have partially addressed through functional assays in this study.

Regarding the specificity of ribosomal gene expression in cluster 4, we analyzed the distribution of ribosomal genes expressed across all clusters, as shown in Supplementary Figure S1-J. This analysis demonstrates that cluster 4 is specifically enriched in ribosome-related genes, reinforcing its characterization as a transcriptionally active population. Given that ribosomal gene expression is a key feature often associated with proliferative or metabolically active cells, these findings support our initial interpretation that cluster 4 may represent an undifferentiated or progenitor-like population.

We acknowledge the reviewer’s suggestion to include a DotPlot to further illustrate the specificity of these genes in cluster 4. However, we believe that Supplementary Figure S1-J already effectively demonstrates this enrichment by presenting the percentage of ribosomal genes per cluster. A DotPlot representation would primarily convey the same information in a different format, but without providing additional insight into the specificity of ribosomal gene expression within cluster 4.

(2) The lineage analysis is highly speculative and based on weak evidences. Initiating the hemocyte lineage to C4 is based on rRNA expression levels. C6 would constitute a better candidate, notably with the expression of PU-1, ELF2 and GATA3 that regulate progenitors differentiation in mammals (doi: 10.3389/fimmu.2019.00228, doi:10.1128/microbiolspec.mchd-0024-2, doi: 10.1098/rsob.180152) while C4 do not display any specific transcription factors (Figure 7I). In addition, the representation and interpretation of the transcriptome dynamics in the different lineages are erroneous. There are major inconsistencies between the data shown in the heatmaps Fig7C-H, Fig S10 and the dotplot in Fig7I. For example, Gata3 (G31054) and CgTFEB (G30997) illustrate the inconsistency. Fig S10C show GATA3 going down from cluster 4 to cluster 6 while Fig 7I show an increase level of expression in 6 compared to 4. CgTFEB (G30997) decrease from C4 to VC in Fig 7F while it increases according to Fig 7I. At last, Figure 7D: the umap show transition from C4 to C5 while the heatmap mention C4 to C6 I believe there is a mix up with Figure 7E.

We sincerely apologize for the inconsistencies noted between the different panels of Figure 7. These discrepancies resulted from using an incorrect matrix dataset during the initial representation. To address this issue, we have fully reprocessed the data and now provide a corrected and improved depiction of gene expression dynamics along the pseudotime trajectory. We are grateful to the reviewer for having help us to correct theses mistakes.

In the revised version, we offer a comprehensive and consistent representation of expression level variations for key genes identified by the Monocle3 algorithm. Supplementary Figure S10 now presents the average expression variation of these significant genes as a function of pseudotime. Based on this dataset, we carefully selected representative genes to construct panels C to H of Figure 7, ensuring coherence across all figures. These updated panels show both average expression levels and the percentage of expressing cells along the pseudotime trajectory, providing a clearer interpretation of transcriptomic dynamics.

We appreciate the reviewer’s helpful feedback regarding our lineage analysis and the suggestion that cluster 6 might be a more appropriate progenitor based on the expression of mammalian-like transcription factors such as PU-1, ELF2, and GATA3. Below, we clarify our rationale for choosing cluster 4 as the root of the pseudotime and discuss the functional implications of the identified transcription factors.

We can hypothesize that clusters 4, 5, or 6 could each potentially represent early progenitor-like states, as these three clusters are transcriptionally close (Lines 539-541). These clusters have not yet been conclusively identified in terms of classical hemocyte morphology, and they appear to arise from ABL- or BBL-type cells. Our decision to root the pseudotime at cluster 4 was motivated by its strong expression of core transcription and translation genes, suggesting a particular stage of translation activity that was not observed for cluster 5 or cluster 6. Cluster 5 and 6 may correspond to a similar population of cells, most probably Blast-Like cells at different stages of cell cycle or differentiation engagement.

Although cluster 6 expresses PU-1, ELF2, and GATA3, which are known regulators of haematopoietic progenitor differentiation in vertebrates, it is essential to highlight that structural homology does not necessarily imply functional equivalence. Moreover, the expression of PU-1, ELF2, and GATA3 does not strictly characterize a population as “undifferentiated” or progenitor-like. Studies such as those by Buenrostro et al. (Cell, 2018) have demonstrated that these transcription factors can remain active in or reemerge during more lineage-committed stages. For instance, PU-1 is essential for myeloid and B-cell differentiation, GATA3 is involved in T-lymphocyte lineage commitment (though transiently expressed in early progenitors), and ELF2 participates in lineage-specific pathways. Thus, their presence does not imply a primitive state but rather highlights their broader functional roles in guiding and refining lineage decisions. Functional annotation of these transcription factors in invertebrate systems remains speculative, particularly as morphological or molecular markers specific to these early hemocyte lineages are not yet fully established. Further functional assays (e.g., knockdown/overexpression or lineage tracing using cells (ABL and BBL) from clusters 4, 5 and 6) will be necessary to determine which hemocyte population harbor progenitor properties and differentiation potential.

To further address the reviewer’s concern, we performed complementary pseudotime analyses by initiating Monocle 3 trajectories from clusters 4, 5, and 6 individually, as well as collectively (4/5/6). These analyses (see attached figure) confirm that the overall differentiation topology remains unchanged regardless of the selected root, consistently revealing two main pathways: one leading to hyalinocytes and the other to the granular lineage (ML, SGC, and VC). This consistency strongly suggests that clusters 4, 5, and 6 represent related pools of progenitor-like cells. Therefore, choosing cluster 4 based on its transcription/translation readiness does not alter the inferred branching architecture of hemocyte differentiation.

We appreciate the reviewer’s suggestions, which have helped us improve our manuscript and clarify our rationale.

**Author response image 1.** Representation of the trajectories obtained from Monocle3 analysis using different pseudotime origins, showing that changing the rooting did not alter the overall differentiation topology. (A) Pathways identified with cluster 4, (B) cluster 5, (C) cluster 6, and (D) cluster 4/5/6 origins.

(3) Concerning the AMP expression analysis in Figure 6: the qPCR data show that Cg-BPI and Cg-Defh are expressed broadly in all fractions including 6 and 7, which is in conflict with the statement Line 473 indicating that SGC (fractions 6 and 7) is not expressing AMP. In addition, this analysis should be combined with the expression profile of all AMP in the scRNAseq data (list available in 10.1016/j.fsi.2015.02.040).

We thank the reviewer for highlighting this point. We acknowledge that the qPCR data show expression of Cg-BPI and Cg-Defh across all fractions, including fractions 6 and 7 corresponding to SGC. However, our conclusion that SGCs do not express antimicrobial peptides (AMPs) was based on a correlation analysis rather than direct detection of AMPs in granular cells. Specifically, the qPCR experiments were designed to measure AMP expression levels in fractionated hemocyte populations relative to a control sample of whole hemolymph. We then performed a correlation analysis between AMP expression levels and the proportion of each hemocyte type in the fractions. This approach allowed us to infer a lower expression of AMP in granular cells, as reflected in the heatmap presented in Figure 6.

Regarding the suggestion to integrate AMP expression profiles from scRNA-seq data, we wrote that the limited sequencing depth of our scRNA-seq analysis was insufficient to accurately detect AMP expression Ligne 472-473 → “However, due to the limited sequencing depth, the scRNA-seq analysis was not sensitive enough to reveal AMP expression.”. Additionally, many of the known AMPs of *Crassostrea gigas* are not annotated in the genome, further complicating their identification within the scRNA-seq dataset. As a result, we were unable to perform the requested integration of AMP expression profiles from scRNA-seq data.

(4) The transcription factor expression analysis is descriptive and the interpretation too partial. These data should be compared with other systems. Most transcription factors show functional conservation, notably in the inflammatory pathways, which can provide valuable information to understand the function of the clusters 5 and 6 for which limited data are available.

We appreciate the reviewer’s suggestion to compare the identified transcription factors with other systems. However, since we did not perform a detailed phylogenetic analysis of the transcription factors identified in our dataset, we refrain from making assumptions about their functional conservation across species. Our analysis aims to provide a descriptive overview of transcription factor expression patterns in hemocyte clusters, which serves as a foundation for future functional studies. While transcription factor profiles may provide insights into the potential roles of clusters 5 and 6, assigning precise functions based solely on bioinformatic predictions remains speculative. Further experimental validation, including functional assays and evolutionary analyses, would be necessary to confirm the roles of these transcription factors, which is beyond the scope of the present study.

Minor comments

Line 212-213: the text should be reformulated. In the result part, it is more important to mention that the reannotation is based on conserved proteins functions than to mention the tool Orson.

We have reworded this section to emphasize that the updated annotation is *function-based*, using Orson primarily as the *bioinformatics tool* for improved GO annotation. We now place the emphasis on the conserved protein functions underlying the reannotation. Lines 212-215 : “Using the Orson pipeline (see Materials and Methods), these files were used to extract and process the longest CDSs for GO-term annotation, and we then reannotated each predicted protein by sequence homology, assigning putative functions and improving downstream GO-term analyses.”

Figure 2: I would recommend homogenizing the two Dotplot representation with the same color gradient and representing the gene numbers in both case.

We appreciate the reviewer’s suggestion to improve the clarity and consistency of Figure 2. In response, we have homogenized the color gradients across the two DotPlot representations and have included gene numbers in both cases to ensure a more uniform and informative visualization.

Table 2: pct1 and pct2 should be presented individually like in table 1

We now present these columns separately (pct1, pct2) as in Table 1, so readers can compare the fraction of expressing cells in each cluster more transparently.

Line 403-414: how many cells were quantified for the phagocytic experiments ?

We have added the exact number of cells that were counted to determine phagocytic indices and the number of technical/biological replicates. Line 411, the text was modified : “Macrophage-like cells and small granule cells showed a phagocytic activity of 49 % and 55 %, respectively, and a phagocytosis index of 3.5 and 5.2 particles per cell respectively (Fig. 5B and Supp. Fig. 7B), as confirmed in 3 independent experiments examining a total of 2,807 cells.”

Line 458: for copper staining, how many cells and how many replicates were done for the quantification ?

We have specified the number of hemocytes and number of independent replicates used when quantifying rhodanine-stained (copper-accumulating) cells. Line 458 the following text was added : “and a total of 1,562 cells were examined across three independent experiments.”

Line 461: what are the authors referring to when mentioning the link between copper homeostasis and scRNAseq?

Single-cell RNA sequencing (scRNA-seq) analysis revealed an upregulation of several copper transport– related genes, including G4790 (a copper transporter) with a 2.7 log2FC and a pct ratio of 42, as well as the divalent cation transporters G5864 (zinc transporter ZIP10) and G4920 (zinc transporter 8), specifically in cluster 3 cells identified as small granule cells. These findings reinforce a potential role for this cluster in metal homeostasis.

We modified lines 462-467 as : “ These results provide functional evidence that small granule cells (SGCs) are specialized in metal homeostasis in addition to phagocytosis, as suggested by the scRNA-seq data identifying cluster 3. Specifically, single-cell RNA sequencing revealed an upregulation of copper transport– related genes, including G4790 (a copper transporter) with a 2.7 log2FC and a pct ratio of 42, reinforcing the role of SGCs in copper homeostasis (see Supp. File S1).”

Line 611: it would be nice to display the enrichment of the phagocytic receptor in cluster 3 (dotplot or feature plot) to illustrate the comment.

We appreciate the reviewer’s insightful suggestion regarding a more comprehensive analysis of phagocytic receptors. While a full inventory is beyond the scope of this study, we acknowledge the value of such an approach and hope that our findings will serve as a foundation for future investigations in this direction.

Although we have highlighted certain phagocytic receptors (e.g., a scavenger receptor domain-containing gene) in our scRNA-seq dataset, it is beyond the scope of the current study to inventory all phagocytosisrelated receptors in the *C. gigas* genome, which itself would be a substantial undertaking. Moreover, singlecell RNA sequencing captures only about 15–20% of each cell’s mRNA, so we inherently lose a significant portion of the transcriptome, further limiting our ability to pinpoint all relevant phagocytic receptor genes. Adding more figures to cover every candidate receptor would risk overloading this paper, thus we focus on the most prominent examples. A promising approach for more exhaustive analysis would involve efficiently isolating granulocytes (e.g., via Percoll gradient) and performing targeted RNA-seq on this cell population to thoroughly explore genes involved in phagocytosis.

Line 640-644: the authors mentioned that ML may be able to perform ETosis based on the oxidative burst.

This hypothesis requires further evidences. Are other markers of ETosis expressed in this cell type?

We agree that additional experimental evidence (e.g., detection of histone citrullination, extracellular DNA networks) is necessary to confirm ETosis in molluscan immune cells. We present ML-mediated ETosis only as a speculative possibility based on oxidative burst capacity as it was shown in different pieces of work that ETosis is inhibited by NADPH inhibitors (Poirier et al. 2014). Nevertheless, the expression of histones in the macrophage-like cluster (cluster 1) reinforces this possibility, as histone modifications play a key role in chromatin decondensation during ETosis.

**Reviewer #2 (Recommendations for the authors):**

Figure 1: In Figure 1B, the cell clusters are named 1 to 7, whereas in Figure 1C they are displayed as clusters 0 to 6. There is a mismatch between the identification of the clusters.

We thank the reviewer for identifying this inconsistency. The cluster numbering has been corrected to ensure consistency between Figures 1B and 1C.

Figure 2B: the font size could be increased for greater clarity.

We thank the reviewer for this suggestion. The font size in Figure 2B has been increased to improve clarity and readability.

Line 221: "Figures 2B, C and D" appears to refer to Figure S2 rather than the main Figure 2.

The text has been corrected to properly reference the figure.

Line 754: "Anopheles gambiae" should be italicised

We thank the reviewer for pointing this out. "*Anopheles gambiae*" has been italicized accordingly.

Bibliography

Integrated Single-Cell Analysis Maps the Continuous Regulatory Landscape of Human Hematopoietic

Differentiation. Buenrostro, Jason D. et al. Cell, Volume 173, Issue 6, 1535 - 1548.e16

Antimicrobial Histones and DNA Traps in Invertebrate Immunity

Poirier, Aurore C. et al. Journal of Biological Chemistry, Volume 289, Issue 36, 24821 - 24831
